# The Baltimore College of Dental Surgery

**Published:** 1893-04

**Authors:** 


THE BALTIMORE COLLEGE OF DENTAL SURGERY.—The fifty-third annual commencement was held at Ford's Opera House, March 20th, 1893, at 2 P. M. The announcement of the graduates was made by Prof. R. B. Winder, M. D., D. D. S., Dean. The address to the graduates was delivered by Rev. H. Allen Tupper, D. D. The class oration was delivered by Robert F. Taylor. of Canada. Prof. R. B. Winder conferred the degree of Doctor of Dental Surgery upon the following gentlemen :

Maryland—Harry C. Griffith, Chas. A. Krantz, Luther M. Parsons, Frank W. Shegogue.

North Carolina—John W. Thompson.

Pennsylvania—W. H. Gregg, P. Edward Harbinson, B. F. Wendell.

New York—Chas. S. Hoose, Albert C. Hays, F, Dwight Joy, John Wood.

Vermont—Claire G. Hamilton, Andrew W. Soule.

Texas—Edward S. Rinehart, Milton S. Merchant.

Wisconsin—Francis H. Mulholland.

Canada—R. Franklin Taylor.

Germany—G. A. Hahn.

Roumania—Joseph Krainik.

The following prizes were awarded to members of the graduating class by Prof. M. W. Foster and Dr. B. Myer:

A diamond medal to Milton S. Merchant, whose grading was 59 out of a possible 60.

Gold medals—G. H. Hahn and Luther M. Parsons, whose grading was 54¼. William H. Gregg received 54, Francis H. Mulholland 63¼, Edwin S. Rinehart 53 and Joseph Krainik 52½, and each received honorable mention.

Operative and mechanical, Joseph Krainik; very honorable mention, J. W. Thompson.

Bridgework and essay on orthodontia, Harry C. Griffith.

